# Engineering microrobots for targeted cancer therapies from a medical perspective

**DOI:** 10.1038/s41467-020-19322-7

**Published:** 2020-11-05

**Authors:** Christine K. Schmidt, Mariana Medina-Sánchez, Richard J. Edmondson, Oliver G. Schmidt

**Affiliations:** 1 Manchester Cancer Research Centre, Division of Cancer Sciences, School of Medical Sciences, Faculty of Biology, Medicine and Health, University of Manchester, 555 Wilmslow Road, Manchester, M20 4GJ UK; 2 Institute for Integrative Nanosciences, Leibniz IFW Dresden, Helmholtzstraße 20, 01069 Dresden, Germany; 3 Gynaecological Oncology, Division of Cancer Sciences, School of Medical Sciences, Faculty of Biology, Medicine and Health, Manchester Academic Health Science Centre, University of Manchester, Manchester, UK; 4 St. Mary’s Hospital, Central Manchester NHS Foundation Trust, Manchester Academic Health Science Centre, Level 5, Research Floor, Oxford Road, Manchester, M13 9WL UK

**Keywords:** Cancer therapy, Targeted therapies, Drug delivery, Nanotechnology in cancer

## Abstract

Systemic chemotherapy remains the backbone of many cancer treatments. Due to its untargeted nature and the severe side effects it can cause, numerous nanomedicine approaches have been developed to overcome these issues. However, targeted delivery of therapeutics remains challenging. Engineering microrobots is increasingly receiving attention in this regard. Their functionalities, particularly their motility, allow microrobots to penetrate tissues and reach cancers more efficiently. Here, we highlight how different microrobots, ranging from tailor-made motile bacteria and tiny bubble-propelled microengines to hybrid spermbots, can be engineered to integrate sophisticated features optimised for precision-targeting of a wide range of cancers. Towards this, we highlight the importance of integrating clinicians, the public and cancer patients early on in the development of these novel technologies.

## Abstract (web summary)

Microbot delivery devices are the latest development in attempts to overcome the systemic toxicity associated with classical chemotherapy. Here, the authors review the recent progress in the field with a focus on the clinical translation and potential of the research and give a future perspective on this topic.

## Introduction

**Fig. 1:**
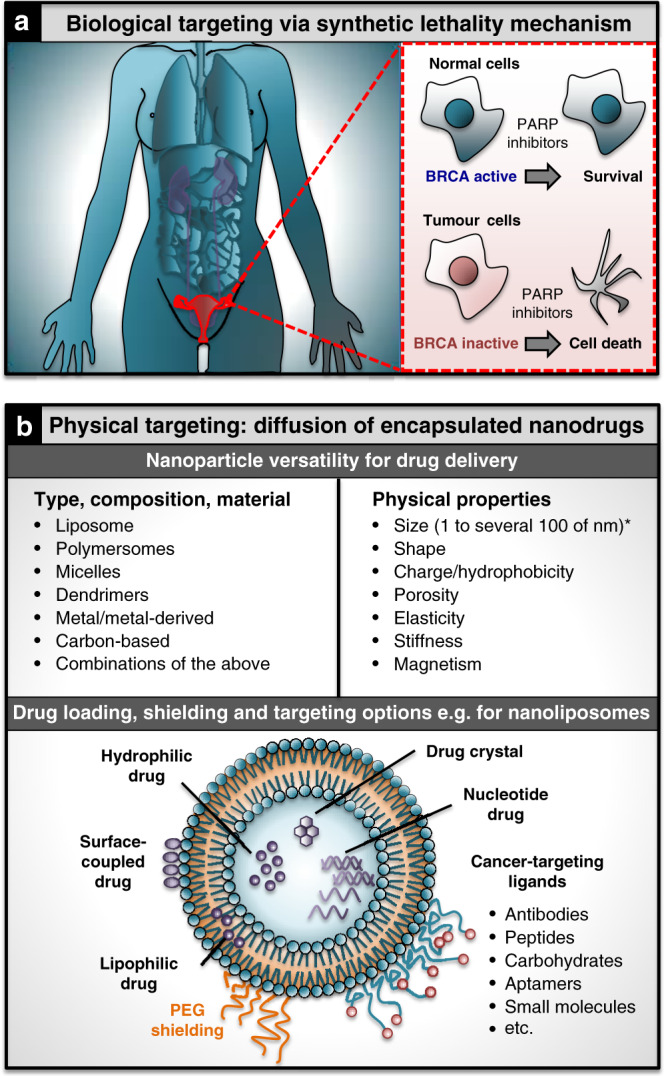
Different approaches towards targeting of cancer over normal cells. **a** Biological targeting: example of targeted killing of cancer cells through synthetic lethality by exploiting genetic differences in the BRCA genes between normal and cancer cells using conventional small-molecule drugs (PARP inhibitors). **b** Physical targeting: engineering drug-delivery systems that can reach and deliver cargo to cancer over normal cells, based on passive/targeted diffusion of nanoparticles that can vary in their make-up (organic and inorganic materials), and display a wide range of physical properties (top). Drug loading, targeting and shielding of nanoparticles can be achieved in distinct ways, as illustrated for nanoliposomes as a key example (bottom). Analogous options exist for other nanoparticle types but require different engineering methods depending on the underlying material, e.g., to allow for surface functionalisation with targeting ligands^2^. *PEG* polyethylene glycol. *Strictly speaking nanoparticles are defined by the International Union of Pure and Applied Chemistry (IUPAC) as particles of any shape ranging in dimension from 1 to 100 nm, but structures up to several 100 nm in size are commonly included.

Systemic chemotherapy is largely unspecific and targets cancers as well as certain normal tissues. Increased efforts are therefore being spent on designing more specific treatments to overcome systemic toxicity issues. To overcome low targeting of conventional treatments, two major strategies exist that aim to achieve tumour-specific effects: systemic drugs that only affect cancer cells, and drugs that are specifically delivered into the tumour cells and/or their microenvironments. The first strategy exploits altered cellular characteristics of cancer cells that facilitate their tumourigenesis. These aberrations can make cancer cells more dependent on cellular pathways that, when targeted, lead to their death, while causing only minor damage to normal cells, a concept known as synthetic lethality (Fig. 1a)^1^. Some of these strategies are now clinically approved^1^. However, they are beneficial still to only a relatively small percentage of cancer patients and limited by tumour heterogeneity as well as drug resistance. Hence, there is a need for approaches towards the second strategy, such as encapsulation of drugs into nanoparticles (Fig. 1b), which can increase drug half-life and tumour targeting^2^. However, success of such nanomedicines has been limited, most likely owing to their dependency on diffusion and the patient’s blood circulation for distribution, thereby limiting their ability of penetrating deep into and accumulating inside cancers, particularly the hard-to-treat hypoxic tumour cores. Microrobots are ideally placed to overcome these limitations of passive nanomedicines. Owing to their ability of moving beyond diffusion these tiny machines, 0.1–100 µm in size, have potential to target cancers more directly and actively. In contrast to passive nanoparticle drug carriers, microrobots can be engineered to penetrate dense healthy and cancerous tissues. Moreover, they can integrate sensing capabilities able to detect aspects of the chemical microenvironment of tumours and help them accumulate there. Microrobots can also be tailored to carry and deliver drugs with high spatiotemporal precision, while protecting their cargo from being diluted by body fluids^3,4^.

Here, we take stock on the promises and challenges of different microrobot classes. We explain how distinct engineering approaches ranging from genetic engineering to chemical and physical fabrication can be applied to different microrobot types to optimise their control and functionality. Moreover, we highlight the importance of early dialogues with clinicians, members of the public and patients. Such dialogues, when pursued carefully and through qualified medical practitioners, are critical for informing future developments to maximise success and fast-tracking of engineered microrobots into the clinic, as well as to facilitate patient uptake and compliance.

## Challenges for physically targeting cancers

**Fig. 2:**
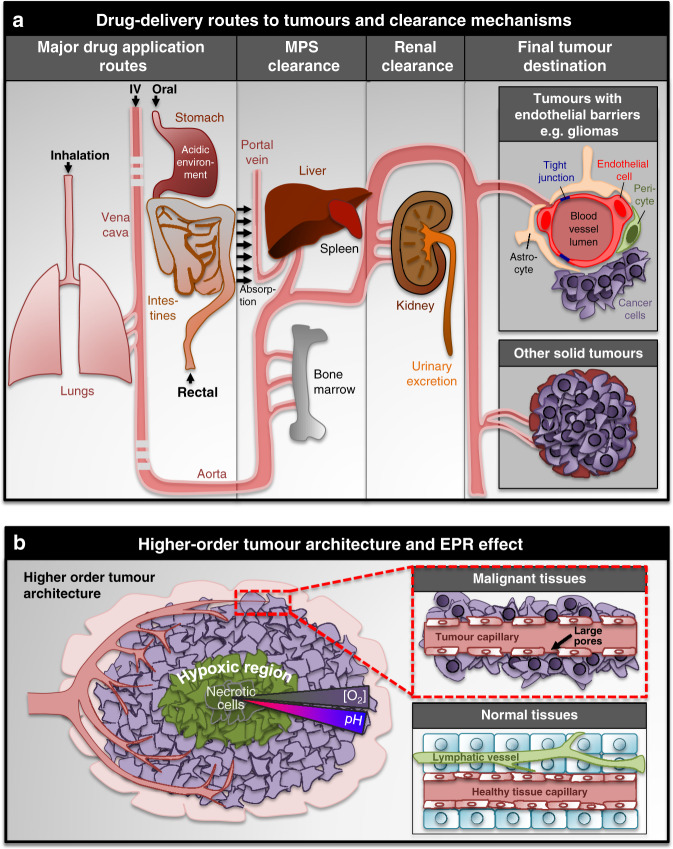
Characteristics of solid tumours and their drug accessibility. **a** Drugs applied by various routes face a variety of long-range targeting challenges such as clearance by the mononuclear phagocyte system (MPS), and organs such as the kidney, liver, lung and spleen, as well as endothelial barriers applicable to tumours developing for instance in the brain or testis. **b** Solid tumours develop a higher-order architecture leading to different characteristics in their core versus their exterior, aspects important for considering/exploiting when developing therapeutic strategies efficient for shorter-term physical tumour targeting towards the hypoxic core. Poor vascularisation inside the core region of the tumour (left) is at the core of the enhanced permeability and retention (EPR) effect (right).

The focus of the review is on physically targeting cancers rather than exploiting altered signalling pathways (reviewed in ref. ^5^). Physical targeting challenges depend on cancer location, type and stage. Tumours can occur at almost any site of the body, including hard-to-reach locations situated deep inside the body, and so-called sanctuary sites, for instance, behind the blood–brain barrier that render them invisible to conventional therapies^6^. Long-range and short-range targeting can be distinguished. Long-range targeting is particularly important for drugs applied systemically, when they need to reach tumours via the bloodstream. Systemic circulation poses distinct pharmacokinetic challenges on the drug that depend on its physical and chemical properties. For example, systemically applied drugs smaller than ~5 nm in size (all small-molecule drugs) are prone to renal filtration and clearance, lowering their bioavailability and thus, requiring high application concentrations. Vice versa, therapeutics larger than a few 100 nm in size become less potent in extravasating through the tiny pores of the vasculature, which possess a similar size in diameter, to reach their target tumour tissues^7^. In addition, systemically circulating drugs need to resist the harsh environments of the gastrointestinal system, including acidic conditions in the stomach and/or high concentrations of degrading enzymes. Depending on tumour location, long-range tumour targeting can further be complicated by specialised endothelial barriers, protecting tumours such as the blood–brain and the blood–testis barrier (Fig. 2a)^8,9^. Local drug application, such as intratumoural injection, overcomes the requirement for long-range targeting, but is often linked to considerable discomfort, particularly when the tumour is situated deep inside the body and/or when multiple applications are required. Moreover, surgery has utility only when disease is confined to a limited number of anatomical sites, when these sites can be accessed without causing significant damage to other structures and when patients are fit enough to undergo what can be a significant surgical procedure. Consequently, surgery has a limited role in the relapse setting where disease is often multifocal and is negatively impacting patient’s performance status.

Once a drug has been delivered into the region of the tumour, short-range targeting takes over. Although certain immunotherapy treatments may be excluded from this concept^10^, most anticancer drugs/cargos need to get and remain in close proximity with the cancer cells to kill them efficiently. For liquid blood cancers, short-range targeting challenges are similar for all cells owing to their suspension as single cells inside the vascular system. By contrast, solid tumours develop a higher-order tumour anatomy^11^, leading to different physical and biochemical microenvironments in the interior and exterior compartments (Fig. 2b). The increased requirement of oxygen of proliferating tumour cells is usually met by additional vascularisation to the exterior compartment of the tumour. Poor vascularisation inside the core region of the tumour, however, leads to a highly hypoxic core^12,13^. Low partial oxygen pressures force cancer cells residing in this area to generate energy via anaerobic, glycolytic pathways that lead to lactic acid generation and thus, acidic conditions inside the tumour core^14^. Moreover, owing to increased proliferation, limited space combined with abnormal blood and lymphatic networks increases the internal interstitial fluid pressure inside a tumour as it continues to grow, making it increasingly difficult to penetrate^15^. Short-range targeting of solid tumour cores is hence extremely challenging. Indeed, hypoxic tumour regions have remained resistant to therapies, making efficient drug delivery to these areas one of the biggest challenges in cancer research.

## Nanomedicines for targeting cancer

Successful physical targeting of cancers would have tremendous patient benefits: absence of the drug from tissues other than the cancer would significantly reduce or eliminate side effects, and lower drug concentrations could be administered less frequently. The last few decades have therefore seen tremendous efforts towards developing medicines accumulating in cancer tissues while avoiding normal organs. One exciting approach has been to package small-molecule drugs, typically below 1 nm in size, into larger nanocarriers up to several 100 nm in size, thereby altering their physical and chemical properties and modulating their pharmacokinetic profiles. Encapsulation of the coupled drugs into such nanocarriers offers advantages including longer drug circulation times, higher cargo capability and increased localised delivery^2^. Nanocarriers have potential to improve long-range targeting in different ways. Owing to their increased size, nanoparticles succumb less to renal filtration and subsequent excretion, which applies mainly to particles with hydrodynamic diameters <~5 nm^7^. Reduced filtration should therefore significantly increase the circulation time of nanoparticles. However, the capacity and characteristics of glomerular kidney filtration can be drastically altered in cancer patients undergoing systemic—often nephrotoxic—chemotherapy^16^. Moreover, when nanocarriers circulate the system, they may face removal by the mononuclear phagocyte system (MPS), also known as the reticuloendothelial or macrophage system, a collection of cells located throughout the body, particularly present in the liver and spleen that can recognise and internalise foreign objects up to several μm in size^17^. Recognition by the MPS can be held in check by cloaking nanoparticles with bioinert non-toxic moieties, such as polyethylene glycol (PEG) or biomimetic modifications^2^, which can also be applied to microrobot applications.

One other striking advantage for nanomedicines is their greater propensity to passively target, and accumulate in, tumour tissues through the enhanced permeability and retention (EPR) effect. This phenomenon is based on poor lymphatic drainage of solid tumours combined with leaky tumour vessels that contain larger pores compared with normal vessels (Fig. 2b). The EPR effect is applicable to particles with long circulation times, ranging in size from ~5–200 nm. These drug carriers escape renal filtration as well as accumulation in the liver, spleen and lung (increasingly relevant to particles >~200 nm in size), while being capable of extravasating through leaky tumour vessel pores (up to ~800 nm). Overall, for drugs requiring circulation through the vasculature, particles in the intermediate size range appear to be the most suitable for overcoming biological filters as well as for being retained in the tumour. The EPR effect further depends on the tumour context and its level of vascularisation as well as the presence of permeability factors, which can considerably vary between different types^7,15,18,19^. To increase the efficiency of systemic nanoparticle applications, the patient’s own physiology has been targeted to improve the EPR effect with antiangiogenic or extracellular matrix-modifying treatments^15^. The EPR effect has formed the main rationale for using nanoparticles as drug carriers to overcome long-range tumour-targeting challenges, and accordingly, in addition to size many physicochemical properties, including shape, surface charge and deformability/degradability, have been explored to maximise the effect^7^. Moreover, nanocarriers can be coupled with targeting ligands including peptides, proteins, antibodies or aptamers^2^. The ligands recognise surface markers on cancer cells and can increase the active short-range targeting abilities of nanocarriers. Despite this promise, evidence is emerging that typically only ~0.7% of administrated nanoparticles reach solid tumours^20^. These drawbacks significantly limit the clinical effectiveness of current nanocarriers, explaining the low numbers of nanomedicines that have been approved as cancer treatments to date^20^.

## Three highly diverse microrobot classes

As a complementary approach to nanocarriers, microrobots have great potential to improve long- and short-range tumour targeting as they combine the advantages of previous nanomedicines in terms of drug protection, selectivity, and biocompatibility, with active motion^3^. Here, we define microrobots as motile microsystems engineered biologically (genetically), chemically and/or physically to exploit their actuation for a specific purpose. Further engineering of these microrobots can integrate additional robotic features such as sensing, tracking and motion control. Cancer-fighting microrobots can be split into three major classes that can be distinguished based on their make-up and source of propulsion: (1) cellular microrobots (biologically actuated) that exclusively consist of cell-made components, and are precision-engineered to exhibit anticancer effects^21^, (2) synthetic microrobots^22^ (chemically and/or physically actuated) that contain only man-made materials, structures and components, and (3) hybrid microrobots, consisting of both artificial and cell-made components that can be propelled by biological or artificial means (usually biologically actuated). Common to all microrobot classes is their active motion, which is attractive for increasing penetration into, and accumulation in, tumours/tissues, as has been illustrated for cells, such as bacteria, immune cells and sperm, and their engineered versions in hybrid microrobots^23–26^. Enhanced tumour penetration also represents an exciting research goal for synthetic microrobots with promising in vivo results emerging^27,28^. Moreover, active motion has potential to increase cellular uptake of delivery vehicles, which has been demonstrated in normal and cancer-relevant cellular contexts based on different actuation mechanisms as well as microrobot size^29–35^.

**Fig. 3:**
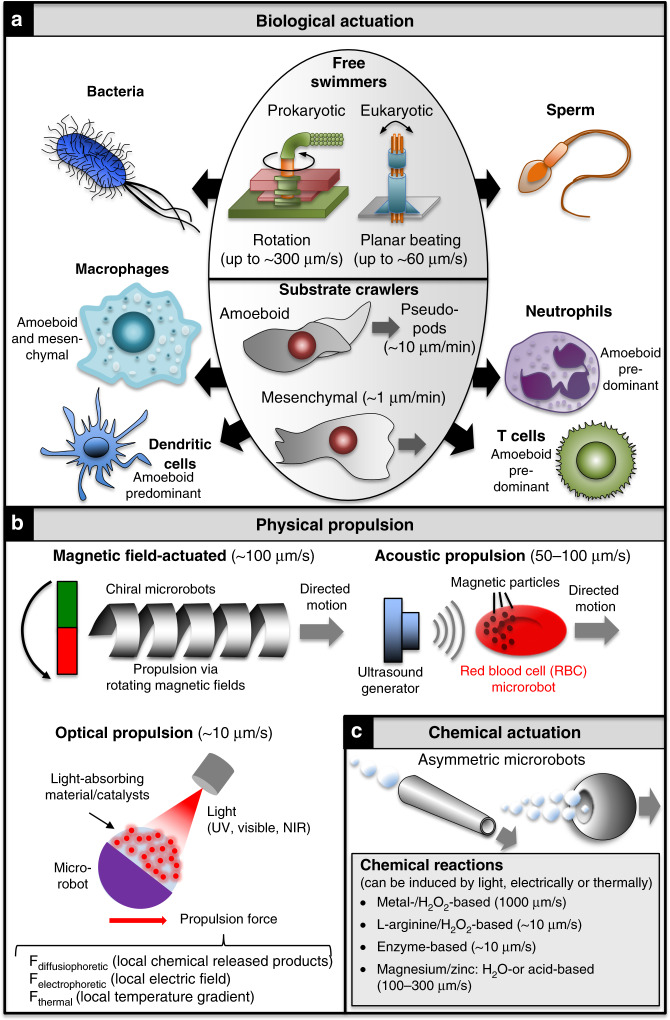
Mechanisms of actuation for microrobots. Key examples for **a** biological migration modes, **b** physical propulsion and **c** chemical actuation.

Biological actuation has independently evolved in prokaryotes and eukaryotes. Bacterial flagella rotate in a propeller-like fashion and can achieve swimming speeds of 300 μm/s (150 body lengths/s; Fig. 3a). First systematic investigations of bacterial therapies date back to the 19th century, when Dr. William B. Coley (1862–1936) employed bacteria to treat malignant tumours leading to cancer regression in some patients but causing fatalities in others owing to the infectiousness of the bacteria and a lack of engineering methods to counteract the toxicity^36^. The development of sophisticated genetic engineering approaches combined with the ability to sequence whole genomes fuelled an era of ‘synthetic biology’ in the early 2000s and contributed to reviving bacterial therapies in recent years by for instance programming their death after use, or making them more stable and effective^37–39^. Moreover, the advent of CRISPR-Cas9 gene editing has extended genetic engineering to higher eukaryotic systems including human cells^40^. Motility has also evolved in eukaryotic cells such as sperm. Rather than rotating, sperm flagella beat in a planar or helical fashion, allowing them to swim up to ~60 μm/s (Fig. 3a)^41^. Other means of biological actuation include amoeboid migration mediated by pseudopodia (false feet; ~10 μm/min) and mesenchymal migration (<1 μm/min) (Fig. 3a), but these processes require surfaces on which to crawl^42^. Amoeboid and/or mesenchymal migration are exhibited by various cell types including phagocytes, neutrophils, monocytes/macrophages, T cells, and stem cells, all of which are being explored towards improved cancer treatments^43–47^.

Propulsion of synthetic microrobots can be achieved through physical or chemical means. Physical actuation can be mediated by varying external physical fields, for example, using magnetic fields^48–50^, ultrasound^51–53^ or light^54^, as well as light-induced thermogradients^27^. Varying physical fields can be used for microrobot propulsion or guidance. By employing rotating magnetic fields for instance, chiral microrobots can be propelled in three dimensions achieving velocities up to hundreds of μm/s, whereas static magnetic fields can be used to orientate self-propelled microrobots. Structures actuated in this manner include segmented nanowires and helices based on ferromagnetic materials such as nickel, iron and cobalt (Fig. 3b)^34,55–57^. Chemical actuation is based on catalytic reactions converting chemical fuel energy into independent mechanical motion. Microrobots of this type typically include a catalyst in their make-up that reacts with fuels in the surrounding medium, resulting in gas bubbles or chemical subproducts expelled from the microrobots. Propulsion can be achieved by one-sided bubble expulsion from asymmetric catalytic microrobots. Alternatively, in ‘two-faced’ Janus-like microrobots the catalyst is constrained to one side of the microrobot, leading to asymmetric fuel consumption, tension or light absorption that induce gradients of chemicals, ions, or heat that propel the microrobot forward. Asymmetric nanorods or nanowires^58,59^ as well as tubal^60,61^, spherical^62,63^ and bowl-like polymersome^29,64,65^ structures have been actuated accordingly (Fig. 3c). A plethora of chemical microrobots has been engineered based on catalysts, such as palladium (Pd), platinum (Pt) or silver (Ag). Recently, chemical and physical actuation have been combined for enhanced control and motility^66^. Although these microrobots are the most powerful ones in vitro in terms of propulsion performance (speeds up to 1000 μm/s; three orders of magnitude above Brownian motion), this has been challenging to reproduce in vivo, as these microrobots require high concentrations of toxic fuels including hydrogen peroxide and organic solvents incompatible with life (reviewed in refs. ^3,67–69^). Excitingly, recent advances have led to the development of chemical microrobots biocompatible with cancer-relevant physiological environments^70–74^. Thus, magnesium- and zinc-based microrobots have been developed that can convert acid/water to hydrogen applicable for certain cancer-relevant contexts/therapies, such as hydrogen chemotherapy, and for treating certain gastrointestinal cancers^28,71,74–77^. Moreover, enzymes such as glucose oxidase^78^, urease^79^ and catalase^61^ can be used as catalysts instead of metals, together with fuels like glucose and urea to reduce toxic fuel concentrations. Finally, a self-destroying microsystem capable of propelling at speeds of ~10 μm/s has recently been engineered that is driven by, and produces, nitric oxide, which is associated with a variety of functions including anticancer activity^80^. Moreover, there are potential concerns over gas bubble toxicity that can be overcome if the bubbles are released into the exterior. Chemically actuated microrobots are thus particularly attractive for targeting malignancies arising in body regions connected to the external environment, such as the gastrointestinal tract, demonstrating how the nature of the microrobot can direct its use towards a select range of cancers.

In the past decade, hybrid microrobots have emerged as a new exciting class of microrobots aimed at combining advantages of cellular and synthetic microrobot components^81–83^. Mostly, these microrobots take advantage of the strong motor abilities of cells, their sensing, processing and tactic migration abilities^84^, and their capability of functioning in complex in vivo environments^81^. By combining them with nanomaterials and other synthetic structures, their targeting and killing efficacies can be enhanced. Alternatively, some immobile cells attractive as drug carriers such as red blood cells, have been made motile by equipping them with magnetic structures for their guidance while being propelled via ultrasound waves^85,86^. A wide range of hybrid microrobots or cell-based materials, such as bacteria^87^, sperm^25,26^, white and red blood cells^88–95^, raphides (needle-shaped calcium oxalate crystals isolated from the *Dracaena sp*. plant)^30^, hair^96^ and *Spirulina* algae^97,98^, have been coupled to different types of synthetic components to generate multifunctional hybrid microrobots optimised for their anticancer efficiency. Owing to the large variety of cellular and synthetic microrobots, and the multitude of nanomedicines that have been developed using different materials, structures, coatings and functionalisations, hybrid microrobots can be combined in a plethora of configurations allowing their tailoring to specific applications defined by the nature of the targeted cancer and its microenvironment.

## Microrobots for long-range cancer targeting

Different long-range challenges apply to microrobots depending on tumour location and the most appropriate administration route. Systemic application is attractive to target most solid tumours throughout the body, haematological malignancies and circulating metastatic cells, whereas cancers located in the gastrointestinal or reproductive tract can also be reached orally/rectally or via the vagina with less-stringent size limitations applying to microrobots administered via these routes. Microrobot characteristics like the ones described above for nanoparticles will at least to some extent also apply to systemically circulating microrobots to allow them to escape biological filters and extravasate. In this regard, it is notable that fabrication methods can be limited to certain size regimes. For example, bottom–up self-assembled synthetic microrobots have only recently been extended to small, sub-micrometre regimes optimised for systemic application^29^. Excitingly, first in vivo data for synthetic microrobots in the sub-micrometre size range are emerging, demonstrating that their motion can markedly promote extravasation into tumour tissues^27^. Other application routes, e.g., via the gastrointestinal tract or the reproductive system allow targeting of certain cancers/cancer lesions inside these systems without the need for systemic circulation, thereby avoiding biological filters such as the lung, liver, spleen and kidney. Future systematic research will shed light on how exactly microrobot motility, size, shape and composition interdepend to determine the half-life, biodistribution and long-range cancer targeting efficiencies of different microrobot types in vivo.

### External guidance

External long-range magnetic fields can reach cancers located anywhere in the body, making magnetism an attractive tool for guiding magnetic particles from their application site to the cancer, independently of local cancer stimuli. However, the magnetic field strengths required for this purpose, depend on various parameters including microrobot size, location in the body as well as composition and architecture of the tissue/fluid the microrobots are operating in. For example, smaller microrobots have less magnetisation and therefore require stronger magnetic field gradients for actuation and guidance. Moreover, the more viscous the medium the microrobot operates in, the stronger the field gradient required for overcoming drag forces. For magnetic resonance imaging (MRI), magnetic fields of up to 10 tesla (T) can be used. According to the US Food and Drug Administration (FDA), however, static magnetic field strengths for clinical procedures should generally be limited to 2 T, unless clear evidence of safety can be demonstrated. In this regard, it is notable that magnetic fields can impact on an organism’s physiology, e.g., by boosting its blood circulation and metabolism: using ~70 mT magnetic fields is sufficient to increase blood microcirculation in rats and affect metabolism. Moreover, these fields can alter cytokine production from lymphocytes and macrophages. Alternating magnetic fields or pulsed magnetic fields can reduce the resulting electric currents in conducting tissues, but the frequency of such fields needs to be sufficiently low to avoid damage by excessive heat. We refer readers further interested in the topic to the following articles^99–101^.

A variety of methods have been developed toward magnetic actuation and guidance. Magnetic resonance navigation is based on magnetic field gradient variation and has been employed to steer therapeutic agents in the vasculature, using an MRI scanner^102^. A permanent homogenous magnetic field (1.5 T or higher) enabled magnetisation saturation and steering of mm sized ferromagnetic objects. Soulez et. al. added a gradient coil inside the MRI scanner, generating stronger fields up to 400 mT, which allowed them to actuate smaller objects in the micrometre range. The materials of the microrobots for this methods are critical and should exhibit the best saturation magnetisation values, such as FeCo alloys^103^. Overall, the required field strength to saturate the microrobots is still limited for this method, and applying it in biofluids deep inside the body is not yet possible. Dipole field navigation (DFN) can achieve high field as well as high navigation gradient strengths for whole-body interventions. In this case, large ferromagnetic cores are introduced around the patient inside the MRI scanner. The current drawback of this method is that it interferes with MR imaging, distorting the static field inside the scanner and thus, limiting it to open-loop navigation. However, implemented strategies like programming imaging sequences, or precise positioning of the cores to achieve targeting accuracy can reduce image distortions^104^. Fringe field navigation aims at providing at high pulling force on a magnetic object. This force is typically produced by a strong fringe field generated by the superconducting magnet of an MRI system. In order to provide directionality to the pulling force, the patient needs to be moved robotically in the magnetic fringe field outside the scanner. Methods like Thin Plate Spline are used to estimate the direction of the magnetic field required to steer an object through a determined path. In this case, fields ~2 T/m are essential to navigate microstructures in smaller vascular networks. A drawback of this method is a high computational load^105^. Electromagnetic actuation-based systems and magnetic particle imaging (MPI)-based platforms represent further developments. MPI is a fast and sensitive imaging modality that enables measurement of the spatial distribution of micro and nanostructures. MPI systems have mm-scale spatial resolution and high temporal resolution. Moreover, a high-gradient field over 1 T/m/µ0 enables nanoparticle-based delivery systems to operate for example deep in the brain. In MPI, the detection threshold of magnetic tracers is less limited by background signals from the host tissue compared with MRI. As a result, image reconstruction methods for MPI can be simpler than for MRI. This method allows real-time and monitoring tasks simultaneously^106^. For a general overview of challenges and limitations associated with different actuation strategies we refer the reader to the following review^107^.

**Fig. 4:**
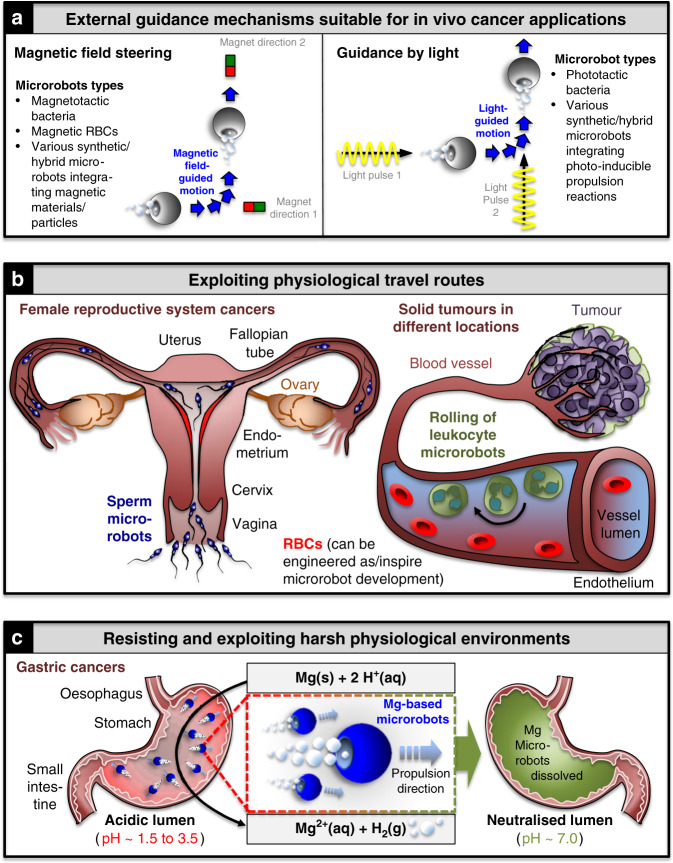
Microrobot approaches for overcoming long-range tumour-targeting challenges. **a** Magnetic field (left) and light (right) steering approaches applicable for in vivo applications. **b** Exploiting physiological travel routes: Left: sperm microrobots in female reproductive tract; tissues relevant for a variety of cancers are highlighted. Right: RBCs and leucocytes travel through vessels that connect and supply tumour tissues. **c** Resisting and exploiting harsh physiological environments: chemically actuated microrobots have a neutralising effect on the acidic environment inside the stomach lumen. *Aq* aqueous, *g* gaseous, *RBCs* red blood cells.

All three microrobot types have been engineered towards external magnetic guidance (Fig. 4a, left). Synthetic microrobots offer a high degree of freedom for generating magnetically responsive microrobots owing to the ability of integrating magnetic components into most synthetic fabrication processes. However, they are still limited regarding their in vivo applicability (see above), and a first magnetically steered synthetic microrobot that can be successfully guided by external means to accumulate inside a cancer is eagerly awaited. Cells can be attached to magnetic particles in different geometrical configurations and cell-to-particle ratios to generate magnetically responsive hybrid microrobots of varying speeds^81,108^. Furthermore, ferromagnetic particles, like iron oxides, can be internalised by eukaryotic cells, such as red blood cells^85,86^, making them amenable to external guidance and acoustic propulsion, or even for hyperthermia-based therapies^109^. Alternatively, magnetotactic bacteria contain naturally synthesised magnetic particles (magnetosomes), allowing them to sense magnetic fields and align their swimming directions along them^110,111^. This intrinsic magnetic responsiveness combined with strong motility (e.g., 300 μm/s for *Magnetococcus sp*.—MC-1) is attractive and suitable for remote control by external magnetic fields as well as by intrinsic tumour-tactic stimuli^112^. Indeed, in a recent landmark study, magnetic guidance led to significantly enhanced tumour accumulation of peritumourally injected MC-1-based hybrid microrobots in living mice compared to non-guided MC-1 bacteria^87^. Recently, magnetic particles have also been produced in *Escherichia coli*, extending this approach to a model system with a wide range of synthetic biology tools available^113^. Also, photocatalytic reactions can be used for actuation and external guidance mechanisms (Fig. 4a, right)^54,114,115^. Although these hybrid microrobots can be successfully guided in vitro, they have not yet been tested for cancer or other treatments in vivo. Collectively, these findings highlight external steering of microrobots as a promising method for overcoming one of the biggest challenges associated with cancer targeting, albeit not without its own challenges (see above, and ‘Outlook’ section below).

### Exploiting and mimicking physiological travel routes/behaviour

Some cells have evolved to intrinsically target remote regions inside our bodies. Sperm are the perfect example: they are adapted to travelling up the reproductive system, parts of which, such as the fallopian tube, are inaccessible and therefore challenging to examine in clinical settings. Engineering sperm has therefore emerged as a promising strategy to target cancers affecting the female reproductive system, including the cervix, endometrium and ovaries (Fig. 4b, left)^25,26,116,117^. For example, early ovarian cancer precursors are typically small non-vascularised lesions accessible via the lumen of the fallopian tube with no need of penetrating large areas of extracellular tumour matrix. Considering the fallopian tube lumen is ~1 mm in diameter in its narrowest section^118^, microrobots in the medium or even large micrometre range can be explored for targeting these lesions. Larger microrobots are more amenable to deep-tissue imaging, real-time tracking and could thus open up precise spatiotemporal control of single microrobots in vivo in these contexts^119^. Another exemplary physiological behaviour used by some of our body’s own cells is the ‘rolling’ of leucocytes when they travel and navigate through the vasculature. By integrating acoustic and magnetic fields synthetic microrobots are being designed based on this behaviour^120–122^. Moreover, spermbots have recently shown promise for cargo delivery through flowing blood^123^. Taken together, these microrobots could be exploited to target metastatic cells inside the vasculature, haematological cancers and/or solid tumours located throughout the body (Fig. 4b, right).

### Resisting and exploiting physiological environments

Several cancers arise in harsh environments inside the body, such as gastric cancers surrounded by acidic digestive fluid (pH 1.5–3.5) inside the stomach. Most drugs in this environment require proton pump inhibitors for neutralisation. By contrast, magnesium and zinc-based chemical microrobots are perfectly adapted to this environment. They cannot only resist the acid but use the protons to generate hydrogen bubbles as a locally supplied fuel for propulsion, resulting in simultaneous neutralisation of the acid (Fig. 4c). These engineered microrobots have recently made it into in vivo studies in mice to treat stomach infections caused by *Helicobacter pylori*, allowing first studies of synthetic microrobot distribution, retention, efficacy and toxicity in living organisms^71,75–77^. Encouraged by these results, a first microrobot pill has been developed^124^. It will be exciting to see how applications of these and related microrobots^28^ will be developed further towards targeting and treating cancers inside the gastrointestinal tract, and how size, shape and composition of microrobots can be fine-tuned to create microrobots optimally suited for this purpose. In addition, bladder cancers are attractive for providing high concentrations of local fuel, i.e., urea, which is present in the bladder and can be exploited for developing urease-driven synthetic cancer cell-targeting microrobots^35^. Other physiological environments that have been explored for tailored microrobot applications include the vitreous body of the eye, attractive to treating eye cancers^125^.

## Microrobots for short-range targeting of tumours

Once microrobots have reached the tumour region, short-range targeting is crucial to maximise tumour penetration, accumulation and retention. In contrast to the physical interactions mediated by magnetic field guidance, chemical interactions act in short-range and can be exploited to increase short-range targeting of all classes of microrobots albeit by different mechanisms.

### Intrinsic tumouritaxis

**Fig. 5:**
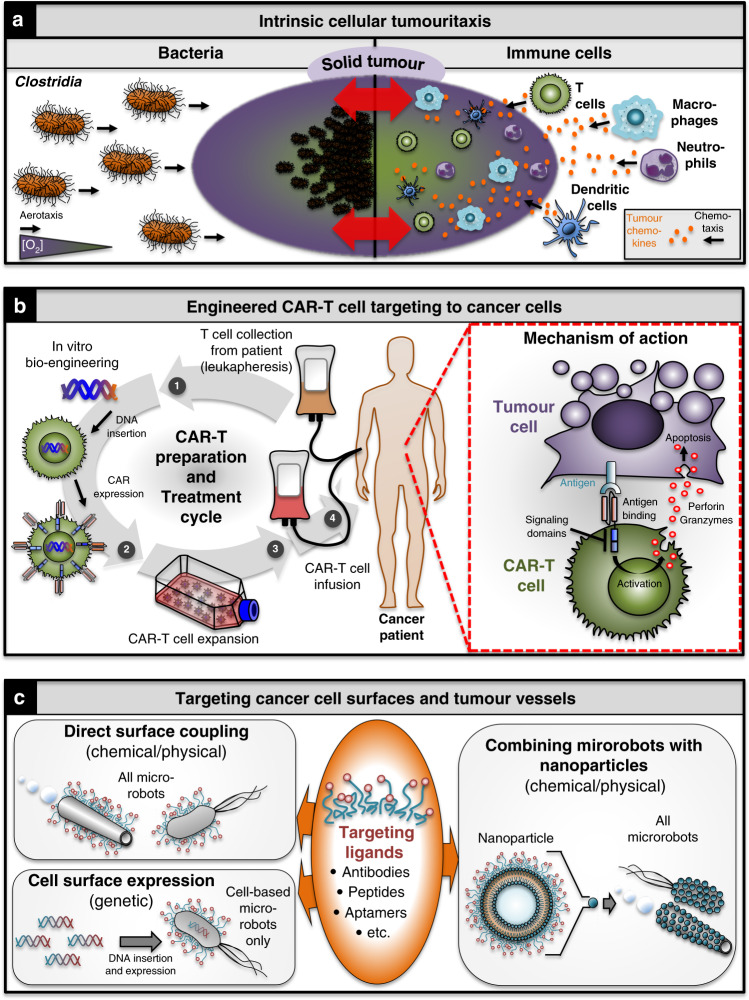
Microrobot approaches for overcoming short-range tumour-targeting challenges. **a** Intrinsic tumouritaxis exhibited by some bacteria, such as *Clostridia*, and higher eukaryotic immune cells. **b** FDA-approved pipeline for engineering CAR-T cells as a personalised cancer medicine (left). CAR-T cells target cancer cells by recognising tumour antigens on their surface that induce downstream TCR signalling, including cytotoxic perforin and granzyme reactions. **c** Targeting tumours via specific surface markers on the cells themselves, or on the vessels that supply the tumour. Using chemical/physical engineering methods, targeting moieties can be coupled directly to the microrobot surfaces or as part of non-motile nanoparticles, as illustrated for nanoliposomes as a key example. Analogous approaches can be applied to other nanoparticles (see also Fig. 1B). Particularly for the nanoparticle-coupling approach, care needs to be taken to limit microrobot size to regimes appropriate for the intended application (see main text for details). For most cellular strategies, the underlying cells can be genetically engineered to express targeting moieties on their surface. *CAR* chimeric antigen receptor, *TCR* T-cell receptor.

Bacterial microrobots possess a unique way of intrinsically targeting tumours in short range by sensing chemical and/or other cues in their environment and moving towards or away from them, a behaviour called taxis^126^. Different tactic behaviours allow bacteria to specifically target solid tumours or regions within them, a behaviour we will refer to in the following as ‘tumouritaxis’. Thus, aerotactic behaviour is key to the tumouritactic abilities exhibited by obligate anaerobic bacteria like *Clostridia*, which allows them to thrive inside the hypoxic cores of solid tumours. Owing to their strictly anaerobic life cycle, *Clostridia* cannot proliferate outside the hypoxic tumour core (Fig. 5a, top left)^127^. Although this reduces their off-colonisation side effects, it also means that intrinsic short-range targeting by *Clostridia* is unsuitable for targeting outer tumour tissues. To overcome this limitation *Clostridia* treatments have been combined with chemotherapeutics capable of targeting outer tumour regions^37,128^. Notably, some bacteria can also accumulate in vivo in mouse tumours independently of chemotaxis and motility, but rather owing to specific immunosuppression in the tumour area^129–131^. However, although motility is not required for tumour colonisation it does enhance it.

Like bacteria, eukaryotic cells such as immune cells exhibit tactic behaviours, allowing them to follow chemical gradients of cytokines to tumours (Fig. 5a, right)^132^. This chemotactic behaviour forms the basis for immunotherapies that boost the natural ability of immune cells to detect and fight cancer cells^44,133,134^. Notably, immune cells are able to interact with cancer cells inside solid tumours as well as in the bloodstream^134^, making them attractive for targeting solid and liquid cancers, as well as metastases, which are the ultimate cause of the majority of cancer-related deaths^135^. In addition to their tumouritactic behaviour, immune cells are able to undergo the process of diapedesis, or leucocyte extravasation, in which cells are able to extravasate the intact vessel wall^136^, and then survive in what is often a hostile tumour microenvironment^137^. These key features are currently being exploited using the process of adoptive T-cell therapy in which autologous therapeutic tumour-infiltrating lymphocytes can be used to treat cancers such as melanomas^138,139^. Alternative strategies include engineering T cells to specifically recognise and activate the immune system against certain types of cancer cells^89–92,140^. For example CAR-T cells, genetically engineered to display chimeric antigen receptors (CARs), have recently been FDA-approved as personalised cancer therapeutics. They show drastically increased efficacy in the clinic, particularly for treating blood cancers refractory to conventional treatment regimens (Fig. 5b)^89,93^. Collectively, the intrinsic abilities of bacteria and immune cells to home in on certain tumours makes them attractive exemplars for how cellular microrobots may be developed into self-directed cancer-battling machines. For the same reason, bacteria and immune cells are attractive for developing targeted cell-based hybrid microrobots that can integrate a range of additional sophisticated features^44,87^.

Equipping synthetic microrobots with intrinsic tactic abilities analogous to those of cellular microrobots remains challenging. Although advances toward photo-, chemo-, and rheotactic (swimming against the flow) systems have been achieved, synthetic microrobots exhibiting true chemotaxis for targeting tumour-specific characteristics are eagerly awaited^141^. However, enhanced activity of synthetic microrobots at/inside tumour sites can be achieved by alternative means, such as using synthetic smart materials able to induce specific tumour microenvironment responses. For example, active motion can be triggered specifically in the acidic environment of cancer cells representative of the lactic acid accumulation in rapidly growing tumours. Similarly, H_2_O_2_ produced by cancer cells under oxidative stress is attractive as a locally provided fuel for synthetic microrobots^142^.

### Targeting cancer cell surfaces and tumour vessels

As a result of mutations and other genetic/epigenetic aberrations, cancer cells and their microenvironments acquire chemical and physical characteristics that differ from normal cells. Specifically, they express cell surface proteins that can be divided into tumour-associated antigens, which are normal proteins being abnormally expressed by the cell, and neoantigens which are novel, abnormal proteins. In contrast to tumour-associated antigens, neoantigens represent unique targets as they are only expressed by the tumour cells, thus minimising the off-target effects seen with many other conventional therapies^143–146^. These attributes can be exploited for cancer targeting based on specific chemical interactions acting in short range. This targeting strategy has been widely used in passive systems, but can also be applied to active systems to further enhance their intrinsic targeting abilities^35^. T cells can, for instance, be genetically modified to express proteins such as CARs on their surface that recognise tumour antigens (Fig. 5c). This strategy led for instance to increased colonisation of *E. coli* at tumour sites and decreased bacterial growth in off-site tissues^147^, highlighting the potential of this approach for increasing the therapeutic index of cell-based microrobots (see also CAR-T cells below). A similar approach can be applied to synthetic microrobots that lack intrinsic targeting abilities. However, because they cannot be genetically modified, the coupling methods differ. Thus, antibodies and other moieties recognising tumour surface markers such as aptamers can be coupled to synthetic microrobots, in a fashion analogous to nanocarriers, to increase their potential of specifically targeting tumours and their microenvironments. These moieties can be coupled directly or indirectly for instance via incorporation into nanoparticles, such as nanoliposomes, that can themselves be glued to the surface of the microrobots to facilitate concomitant drug delivery (Fig. 5c)^87,148^. However, depending on the travelling route to the target tumour, caution needs to be taken to limit microrobot size. For example, systemically administered microrobots needing to extravasate and penetrate deep into solid tumour cores need to remain below the pore radius exhibited by the tumour vasculature (up to ~800 nm) as well as within the dimensions of interstitial extracellular matrix spaces and basement membrane pores (nm to low μm range)^7,149,150^. Importantly, long- and short-range targeting mechanisms have already been successfully combined in hybrid microrobots, leading to increased targeting efficiencies including accumulation in hypoxic tumour regions^87^.

## Microrobot-induced killing mechanisms

After reaching the tumour, microrobots need to be able to eradicate the cancer cells. Two major pathways can be distinguished. We will focus on one that is direct, can be applied to all three microrobot classes and is based on transforming microrobots into drug-delivery vehicles that can load, deliver and release drugs to cancer cells and/or their microenvironments. Some of the approaches below are inspired by nanoparticles, and we refer readers interested in general advantages and challenges associated with these strategies, to the following review^2^. A second pathway relies on microrobots eradicating cancer cells indirectly via their ability to activate or stimulate the patient’s immune system against the targeted cancer. For example, bacteria-based strategies can help boost the overall therapeutic response not only to the originally injected cancer, but also to tumours occurring in other locations of the body^151^. This abscopal effect is particularly attractive for eliminating metastases and establishing a durable systemic anticancer response. Characterising better how different designs of microrobots impact on activating the immune system and how this could further be exploited for microrobot performance is hence an area of active investigation^152,153^. Finally, the propulsion/actuation abilities of microrobots could per se enhance their therapeutic function by mechanically killing cancer cells as previously demonstrated with magnetically actuated microdisks^154^.

### Loading drugs into/onto microrobots

**Fig. 6:**
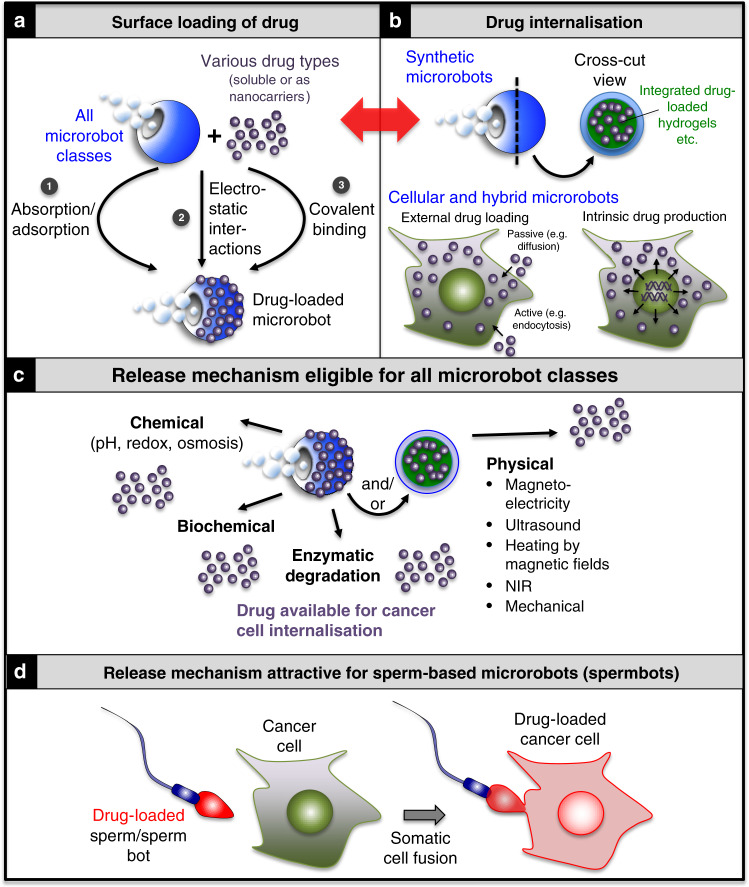
Drug-loading and release mechanisms for the three classes of microrobots: cellular, synthetic and hybrid types. **a** Surface loading of drugs applicable to all three microrobot classes. **b** Drug internalisation via integration of drug-loaded porous materials such as hydrogels into the manufacturing process of mostly synthetic microrobots (top), cellular uptake of external drugs by passive (e.g., diffusion, osmosis) or active (e.g., endocytosis) means (bottom left) as well as drug loading of cellular or cell-based hybrid microrobots through cellular expression/production of therapeutic endogenous compounds such as granzymes/perforin by T cells or genetically engineered drugs such as prodrug enzymes (bottom right). Red arrow indicates overlapping methods for the indicated processes. **c** Release of surface-loaded and/or internalised microrobot drugs can be mediated by chemical, biochemical, enzymatic and/or physical means. **d** Attractive biological drug-release methods include somatic cell fusion of sperm/spermbots. *NIR* near infra-red light.

Therapeutic compounds can be coupled directly or as part of larger nanocarriers in ways depending on the microrobot class and nature of the therapeutic compound. Larger microrobots have potential to be loaded with higher drug doses. However, increasing the number of smaller microrobots can compensate this effect and amount to the same total dosage. The downstream application determines which approach is the most suitable, factoring in considerations such as imaging modalities and external guidance mechanisms applicable to different microrobot size regimes. Most anticancer microrobot applications to date have used small-molecule drugs to perform proof-of-concept demonstrations, such as doxorubicin (DOX)^155^, camptothecin (CPT) or their analogues, that exhibit broad anticancer efficiencies and display autofluorescence to facilitate monitoring of drug loading and distribution. Alternatively, fluorescent molecules such as fluorescein isothiocyanate or methylene blue have been used as model drugs^63^. These compounds can be coupled by absorption, adsorption, electrostatic interactions or covalent binding and loaded onto the surfaces (Fig. 6a) or into the interior (Fig. 6b, top) of microrobots^25,44,156^, analogous to principles established for nanocarriers^157^. Finally, cells can be exploited as drug reservoirs in cellular and hybrid microrobots (Fig. 6b, bottom), such as sperm, neutrophils and red blood cells, where the drug is protected from dilution by body fluids by the plasma membrane^25,26,85,86,95,156^. Some cellular microrobots particularly immune cells can also produce and store endogenous cytotoxic compounds such as perforin and granzymes (cytotoxic proteases), thereby acting as natural therapeutic reservoirs^158^.

### Drug release from microrobots

For drug-loaded microrobots to be maximally effective, they need to be able to release their cargo at the correct time and destination in a controlled way. Several principles towards this aim have been explored including chemical, biochemical, enzymatic and physical means (Fig. 6c). Chemical release has been achieved by integrating pH-, redox- or osmosis-responsive materials into microrobots^64,159,160^. For example, when synthetic drug-loaded nanorobots coated with a pH-responsive polymer were internalised by cells, the polymer dissolved due to the higher intracellular pH, leading to release of the drug followed by high cancer cell-killing efficiencies in vitro^160^. Magnesium and zinc-based synthetic microrobots acting inside the stomach fall into the same class: their embedded cargo is released upon dissolution of the microrobot by the surrounding acidic environment^28,76^. Recently, a drug-loaded stomatocyte-based hybrid microrobot has been developed that integrates redox-responsive disulphide bonds between the polymeric blocks that constitute its scaffold. Drug release occurred upon stomatocyte internalisation by the cell, when glutathione, an antioxidant present in the cellular cytosol, induced the collapse of the stomatocyte and led to the release of the drug into the cell^64^. Drug release can also be induced by a solvent-switch method combined with dialysis. The osmotic shock triggered by this method induces the formation of large pores in the stomatocyte microrobot surface, which then leads to sustained drug release^64^. Biochemical release mechanisms can be exploited when integrating biodegradable materials into microrobots, such as aliphatic polyesters susceptive to hydrolytic degradation. For example, degradation of stomatocytes has been accelerated by lipolytic enzymes, such as intracellular lipases that then trigger the release of the drug from the microrobot^161^. Physical release mechanisms based on magnetoelectricity^162^, ultrasound^163^, heating by magnetic fields, irradiation with near infra-red (NIR) light as well as via mechanical effects represent alternative approaches. For example, microrobots incorporating magnetoelectric materials can change their electric polarisation when stimulated with alternating magnetic fields, which can trigger the release of anticancer drugs in addition to facilitating actuation^162^. Drugs can also be loaded into heat-sensitive materials of microrobots, such as gelatine or other polymers. When integrated with gold nanoparticles, irradiation of such microrobots with NIR light causes the gold particles to absorb the NIR light and convert it into local heat via the photothermal effect, which dissolves or restructures the heat-sensitive gelatine/polymers, thereby releasing the drug^52,60,62^. Importantly, the magnetic, acoustic fields and infra-red light irradiation required for the release mechanisms^52,109,164,165^ represent approaches feasible for in vivo use in humans. In addition, a mechanical mechanism has been employed in vitro to release drug-loaded spermbots coupled to magnetic tetrapod caps into cancer spheroids, to which they were guided using magnetic fields. The flexible arms of the tetrapod entrapping the sperm opened up when the spermbot hit the spheroid, allowing the drug-loaded sperm to penetrate deep into the cancer cell mass^25^. Finally, biological drug-release mechanisms such as cell–cell fusion between cancer cells and cell-based microrobots represent attractive strategies to be explored further for instance for spermbot-mediated drug delivery directly into cancer cells (Fig. 6d)^25,26^.

## Translatability challenges

**Fig. 7:**
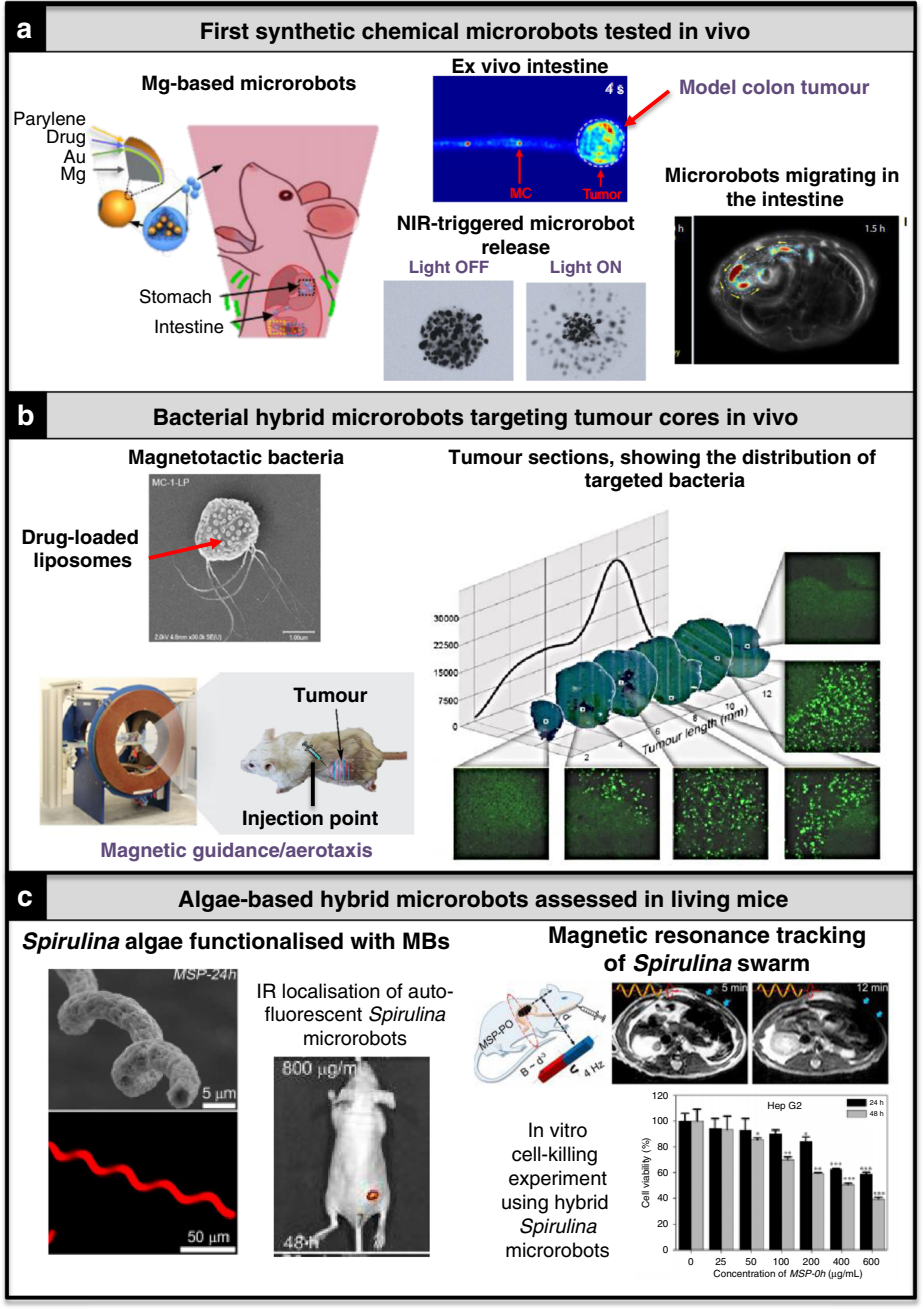
Key synthetic and hybrid microrobot examples for in vivo applications towards cancer treatment. **a** Synthetic chemical microrobots tested in the gastrointestinal system in living mice. Reproduced with permission from ref. ^28^ © AAAS. **b** Hybrid microrobots based on magnetotactic bacteria targeting hypoxic tumour cores. Reproduced with permission from ref. ^87^ © Springer. **c** *Spirulina*-based magnetic hybrid microrobots explored in vivo. *MBs* magnetic beads, *NIR* near infra-red light, *IR* infra-red light. Reproduced with permission from ref. ^98^ © AAAS.

To maximise efforts of microrobots towards clinical translation, it will be crucial to optimise microrobot development to the specific needs of the targeted cancers. Factors to consider are the location of the cancer within the body (what propulsion methods fit best?) and the molecular make-up of the cancer (which drugs will achieve the best killing efficiencies)? To be able to address these questions, microrobots need to be optimised in model systems closely resembling the in vivo conditions of the cancer. Cell-based strategies, particularly bacterial therapies and cellular immunotherapies have been tested most in this regard. It is therefore no surprise that precision-engineered cell-based therapies have progressed furthest to the clinic. For example, CAR-T cells were approved by the FDA in 2017 as the first gene-therapy in the USA, highlighting the tremendous clinical success of a specific type of cell-based therapy (Fig. 5b)^166^. Moreover, various attenuated *Clostridia* and *Salmonella* species have been tested in preclinical cancer studies, and some are being assessed in clinical trials in humans^37,38,167^. These strategies could form the basis for advanced hybrid microrobot therapies in the future. For the younger fields of synthetic and hybrid microrobots, mostly model drugs rather than actual chemotherapeutics have been tested using mainly in vitro 2D or 3D cultures of cancer cell lines, which significantly differ from primary cancer cells. However, studies for synthetic (Fig. 7a)^27,28^ and hybrid microrobots (Fig. 7b, c)^87,98^ in living organisms are increasingly emerging. Important next steps are to extend these studies to other microrobot types and clinical trials. Towards this, it will be key to focus research on patient-representative cancer contexts, such as using ex vivo patient-derived cancer cell cultures^25^, as well as on the most relevant mouse models, and choosing chemotherapeutics best-suited for the targeted cancer not only in its naive form but also after first-line treatment regimens, bearing in mind that clinical trial participants have previously often undergone surgery, chemo-/radiotherapy or immunotherapy. In cases where a previous chemotherapy made use of the same drug that forms part of the microrobot application, this could result in reduced efficiency owing to resistance mechanisms. Moreover, microrobots targeting radiotherapy-treated tumours have to penetrate through extracellular matrix structures of increased density, making it more challenging to reach the hypoxic core.

Another key challenge is the scalability of microrobots, particularly for systemic therapies aimed at treating solid tumours, which require a large number of applied microrobots. Although higher microrobot yields are anticipated compared with passive nanoparticles, biological filters will invariably reduce yields as outlined above. As a comparison, a typical dosage for CAR-T-cell therapies consists of ~10^5^–10^10^ cells/kg^168^. To put these numbers into context: for producing the “chassis” of microrobots, a variety of methods exist. Depending on microrobot size, geometry and make-up, many thousands to millions of structures can be produced in a relatively short time and with high reproducibility. The latter is key because even small alterations in microrobot geometry can lead to malfunction or poor performance. Maskless 2D lithography can fabricate ~6 million asymmetric microtubes a day, e.g., to generate chemically actuated microrobots or hybrid spermbots based on a 4” wafer. This approach can easily be scaled up, and sped up approximately four times if using a mask (own experience). Glancing angle deposition can fabricate ~10^9^ structures by 3D physical vapour deposition, using a 4” wafer^17^. Structures produced by electrodeposition are defined by the shape, size and density of pores inside a membrane onto which organic and inorganic materials are deposited that determine the final microrobot structure. As a rough guideline, this method can produce thousands of structures in a few hours with the precise yield depending on pore density and size of the underlying membrane. Another common method for fabricating complex 3D microrobots is two-photon lithography, yielding ~7 thousand structures per day when running in piezoelectric mode, and ~100 times more in galvanostatic mode (own experience). Strain engineering is based on 2D lithography and deposition methods, and as such is scalable to wafer size. The technique is compatible with conventional microelectronics, thereby offering the possibility of integrating additional functionalities into self-assembled microrobots, e.g., on-board sensors, antennas or microactuators. Finally, chemical synthesis, as a bottom–up fabrication approach, offers perhaps the highest fabrication yield: depending on the reagents and reactors used, vast amounts of structures can easily be generated in gram scale in a few hours.

The more complex the microrobot the bigger the challenge for scaling up, particularly when combining the chassis with additional advanced functionalities, e.g., cell coupling, drug loading, targeting, coating and labelling, which may require considerable optimisation to achieve appropriate stability over time, both during storage as well as after application. This applies particularly for microrobots carrying components such as drugs or catalysts on their surface (Fig. 6a), which could trigger immune responses or lead to proteins from body fluids, particularly the blood, to adhere or degrade the microrobots. Moreover, it is notable, that longevity can be beneficial (e.g., the oxygen consumption by cell-based strategies in the hypoxic areas to create necrosis) or undesirable when it represents a risk of propagation, e.g., for certain cell-based therapies that can lead to systemic toxicity. Manufacturing processes such as stamping, selective material functionalisation, physical ad/absorption of biomolecules etc. allow small-scale sample preparation and optimisation for such strategies with relatively well-established and simple protocols. Automating reactor parameters (steering speed, temperature, etc.) can reduce production times towards scaling up. However, some therapies including those based on adoptive T cells require patient T cells to be extracted from the tumour to then be cultured in large scale and to clinical standard (Fig. 5b). Upscaling is therefore difficult to automate and as a consequence, such treatments are expensive^169^. In many instances, a complete change in and standardisation of fabrication methods may be unavoidable to ensure mass production of homogeneous samples with high batch-to-batch reproducibility. Manufacturing procedures also need to be clearly defined and strictly controlled, so that clean and hygienic microrobots can be generated that fulfil good manufacturing practice (GMP) requirements and are safe to use by humans. In this regard, synthetic and hybrid microrobots are venturing unchartered territory given the current lack of precedence for standardised protocols and appropriate machinery use in industry. Hence, scaling up to GMP standard will be one of the biggest downstream challenges facing these microrobots before they can be approved and licensed for clinical use. Indeed, some trade-offs in the design are likely to be unavoidable. Aspects, such as simplicity of manipulation and synthesis will have to be weighed against biological complexity and logistical challenge to maximise the feasibility of the developed microrobots for downstream translatability.

One of the most fascinating features of microrobots is their amenability to external guidance when integrated with magnetic components. However, controlling magnetic microswimmers in 3D space requires sophisticated infrastructure (e.g., electromagnets that generate rotating magnetic fields or magnetic field gradients), as well as robust control systems to improve microrobot manoeuvring, involving haptic control mechanisms to virtually perceive applied forces during their actuation^170,171^. For the moment, such systems have been mainly used at small scale for in vitro studies, coupled to optical microscopes or ultrasound probes. As a next important step, it will be exciting to extend these efforts to whole organisms by integrating for example electromagnetic actuation to small animal bioimaging systems (e.g., fluoroscopy, ultrasound, infra-red coherence tomography and optoacoustic tomography) to assess feasibility for human trials. Notably, the magnetic actuators can be easily scaled-up but then the imaging and feedback control of the untethered microswimmers become more challenging owing to the current spatiotemporal resolution of ~150 µm in real time when using cutting-edge ultrasound and optoacoustic imaging systems^28,172–176^. In this regard, the increased size of individual microrobots over nanomedicines or using microrobot swarms could provide distinct advantages^119,177–179^. However, microrobot size also represents a limitation, as microrobots need to remain small enough to penetrate and reach tumours efficiently as outlined above. In order to translate magnetic field actuation systems to the clinic, array systems of electromagnetic coils can be scaled up to produce fields in the order of hundreds of mT up to T, sufficient to penetrate the whole body without causing adverse effects. These magnetic systems can be designed as part of current MRI imaging devices, or they could be integrated into robotic arms, able to move along the patient’s body for local actuation of microrobots, while simultaneously allowing their visualisation. Different imaging methods are suitable for subcutaneous and deep-tissue applications, some of them offering real-time tracking with high spatial resolution. The most suitable imaging technique depends on the intended application and nature of the microrobots as outlined above. For example, if microrobots require external intervention for guidance or actuation, real-time tracking is desirable. However, if microrobots are self-propelled, applied close to the target site, and capable of following local chemical or biological cues, less frequent monitoring may suffice to verify microrobot position and functional performance^180^.

Most importantly, all microrobot types need to be assessed regarding their toxicity to ensure that, independently of their make-up, they are safe before using them in the clinic. Hence, they need to be biocompatible^95,181–183^, and amenable for inactivation and/or clearance from the body once they have performed their actions. The nature of the microrobots determines the principles available to achieve this goal. The early bacterial cancer therapies performed by William Coley in the late 19^th^ century highlight the issue of uncontrolled proliferation of bacteria, which is still limiting their clinical use. Thus, integrating self-destruction switches^184^ as well as extending bacterial therapies to probiotic/commensal strains used as food supplements are being explored^151^, although part of the efficiency of some bacterial therapies seems to depend on their intrinsic toxicity. Further understanding of the precise mechanism-of-action of these cellular microrobots is therefore required to address these issues. For magnetic microrobots in particular, one possibility is to remove them using magnets. Indeed, magnetic nanoagents have recently been retrieved from the bloodstream using intravascular magnetic catheters^185^. We envision these findings to pave the way also for safe therapeutic removal of magnetic microrobots. Internal removal of microrobots produced from biodegradable materials represents another strategy, allowing the microrobots to be cleared for example by surrounding enzymes (e.g., hyaluronidases, acrosins, collagenase, trypsin, etc.), and/or local pH- or temperature-dependent processes^64,186,187^, as illustrated recently for instance for stomatocytes^64^. However, remaining subproducts and coating materials could still induce undesired immunoreactions or toxicity effects. Many strategies to overcome these issues have been applied to drug carriers like nanoparticles, liposomes and cellular carriers, which can be implemented. However, there is still a long way to verify the safety application of the most promising reported microrobots in the most appropriate in vitro and in vivo settings. Overall, it will be important to generate longer-term safety profiles and assess the risks and benefits for patients as individuals as well as on a population level^156,188^ following the standard three phases of clinical trials applied to all candidate drugs. However, innovating oncology trial designs to operate more efficiently, implementing early-stage decision-making processes for candidate drugs likely to succeed, and ensuring stakeholders continue to share and discuss their experiences/problems will help the field adapt to an increasing number of drugs targeting specific cancer types versus generic anticancer agents, which will also benefit targeted microrobot strategies^189^.

**Fig. 8:**
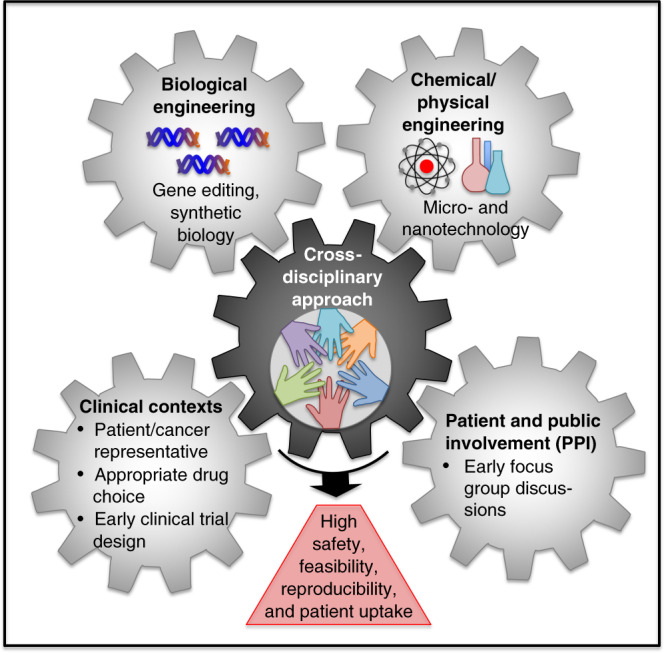
Cross-disciplinary approaches required to maximise translatability and patient uptake of microrobotic drugs in the future. Interactions between basic scientists such as biological, chemical and physical engineers, as well as input from clinicians, patients and the public are critical for further developing microrobots as therapeutic and diagnostic anticancer tools.

Finally, given the new and unconventional aspects of many microrobot types as anticancer agents, it will be critical to get patients and the general public involved at an early stage to contribute to optimal patient uptake and compliance. One example that highlights this point is the potential use of sperm to treat cancers of the reproductive tract^26^, particularly high-grade serous ovarian cancer, which arises inside the fallopian tube and represents a cancer of unmet need with no current curative treatment options available for the majority of patients. We engaged a few patients early on in our work to discuss potential issues such as using partner sperm versus sperm obtained from anonymous donors. Their unanimously enthusiastic responses toward either approach gave us the green light to pursue with our studies, but could have also indicated potential hurdles for patient acceptance down the line, that would have allowed us to adjust our research strategy at an early stage. Therefore, we call on microrobot researchers working in this area to cross-collaborate with basic and clinician scientists as well as involve relevant cancer patients and the general public as early as possible in their studies as soon as basic feasibility has been established (Fig. 8). Close collaboration with clinicians will be crucial in this regard, as they often have focus groups established in their departments that comprise patients and general members of the public to discuss such issues. That way potential challenges and specific needs towards acceptability can be identified and allow researchers to adjust their goals at an early stage. Such dialogues need to be pursued carefully through qualified medical practitioners to avoid unintended consequences on patients grappling with serious and life-threatening diseases such as cancer. The support of dedicated clinicians is also essential for designing appropriate clinical trials (with high-risk groups initially) and emphasising them properly and early on to the public, thereby minimising a common bench-to-bedside hurdle of insufficient patient numbers enroling in clinical trials. In addition, funding bodies and charities are increasingly providing opportunities for helping to integrate patients and the public into their procedures in a careful and controlled way, that is beneficial to all the parties involved.

## Outlook

In conclusion, each microrobot class has its own strengths and weaknesses. Key advantages of cell-based microrobots include tumouritaxis, strong motor abilities in vivo and their amenability to genetic engineering approaches, allowing them to be equipped with functionalities such as directed motion by phototaxis^115^ and chemotaxis^190^. Moreover, cell-based microrobots can be genetically re-wired to respond to extracellular molecules by producing fluorescent or luminescent signals^191^, which makes them attractive for remote in vivo sensing and non-invasive tumour tracking by external imaging^192,193^, features that could become useful also for developing microrobots towards early cancer detection, an area that is increasingly being recognised for its importance^194^. As living and often proliferating materials, cells are however, less well defined and tend to be more challenging to control than synthetic components. Hence, they will generally be more difficult to scale up to clinic standard and to store efficiently. Moreover, there are severe risks of systemic toxicity associated with uncontrollable proliferation of cell-based strategies inside the body. By contrast, synthetic microrobots have the possibility to integrate any kind of material—organic or inorganic—for improved actuation, cargo-release, imaging and sensing tasks^3,170,195–198^ and enhanced controllability. However, potential toxicity effects need to be considered, e.g. using materials with favourable toxicity profiles or dissolvable components, to facilitate clinical progression. Moreover, although first examples of synthetic microrobots have recently reached in vivo settings^27^, the field is still young and future work towards developing microrobots optimised for propelling in applicable biofluids and tissues are required to help extend these successes^116,123^. By combining cellular and synthetic components, almost any functionality can be envisioned in hybrid microrobots matching to the needs of the targeted cancer and its location inside the body. Moreover, as a complementary approach to controlled and localised drug-delivery, physical therapies, such as active photodynamic therapy and hyperthermia, which offer alternative methods, could increase cell-killing efficiency. In photodynamic therapy non-toxic oxygen is converted into reactive oxygen species (ROS) using photosensitisers, which can be embedded into synthetic or biological microrobot components. This concept was recently demonstrated using red blood cells that contained both oxygen and photosensitisers. They were steered to the target location by ultrasound and oriented through external magnetic fields^94^. The transition between non-toxic oxygen into ROS occurred when applying UV-visible light. Although the light source limits its application in vivo, other future stimuli can be envisioned to increase their applicability. Likewise, in hyperthermia therapy target-cells/tissues are exposed to higher than physiological temperatures. Recently, ferromagnetic colloid swarms were steered to cancer cells in vitro using a low-frequency magnetic field, where—under appliance of a high-frequency magnetic field—they induced local heating sufficient to kill the cancer cells^177^. Collectively, it is clear that microrobots hold great promise for oncology, and we envision bright prospects for these tiny machines to efficiently detect and treat a wide range of cancers in the future.
